# Mesalazine-Induced Interstitial Nephritis in a Patient With Ulcerative Colitis

**DOI:** 10.7759/cureus.32488

**Published:** 2022-12-13

**Authors:** José da Cunha Marques, Fernando Nogueira, Catarina Pereira, Ana Carmo Valente, Ana Margarida Ribeiro

**Affiliations:** 1 Internal Medicine, Centro Hospitalar e Universitário de São João, Porto, PRT; 2 Oncology, Centro Hospitalar e Universitário de São João, Porto, PRT

**Keywords:** mesalazine, ulcerative colitis, inflammatory bowel disease, acute kidney injury, acute interstitial nephritis

## Abstract

Acute interstitial nephritis (AIN) corresponds to a decline in kidney function due to an injury induced by drugs (in the majority of cases), infections, and autoimmune disorders. It is characterized by the presence of an interstitial inflammatory infiltrate in the kidney. Here, we describe a case of a man with a previous medical history relevant to ulcerative colitis (UC) who was admitted due to acute kidney injury (AKI) in the setting of AIN secondary to mesalazine.

## Introduction

A 38-year-old Caucasian male with a previous medical history of smoking (11 pack-years), chronic kidney disease (CKD) stage 2 of unknown etiology (with a baseline serum creatinine of 1.5 mg/dL-estimated glomerular filtration rate between 60 and 70 mL/min/1.73 m2) and ulcerative colitis (UC) diagnosed when he was 35 years old, was admitted to the emergency department due to acute kidney injury (AKI) with a serum creatinine of 2.19 mg/dL on routine blood tests. For UC, he was initially medicated with azathioprine, discontinued after hepatotoxicity. In the past three years, he was under mesalazine 800 mg three times per day.

## Case presentation

The patient complained of malaise, asthenia, anorexia, unintentional weight loss (approximately 5% of his body weight), and diarrhea (without blood or mucus) within a month of duration. He denied fever, arthralgias, myalgias, skin rash, changes in urinary output, hematuria, sicca symptoms, ophthalmologic complaints, nausea, vomiting, or abdominal pain. There was neither a history of non-steroidal anti-inflammatory drug abuse nor exposure to proton pump inhibitors, antibiotics, or other drugs and natural products. He denied exposure to contrast agents or recent travel history.

On admission, the patient was hemodynamically stable, apyretic, with preserved diuresis, and without uremic symptoms. The remaining anamnesis and physical examination did not reveal any additional findings. The laboratory workup is displayed in Table 1. Blood samples revealed an elevated C-reactive protein of 44.6 mg/L, AKI with a serum creatinine of 2.24 mg/dL, and mild microcytic hypochromic anemia without leukocytosis. There was no evidence of cholestasis, hyperbilirubinemia, hepatic cytolysis, or hypoalbuminemia. Analysis of serum blood electrolytes did not reveal any changes; lactate dehydrogenase and muscular enzymes were normal. Spot urine test showed discrete leukocyturia of 16.7 cells/mL (normal range < 15 cells/mL), without nitrites, erythrocyturia, casts, or proteinuria. Arterial blood gas analysis did not reveal metabolic acidosis or hyperlactacidemia. Serologies for human immunodeficiency virus (HIV) and hepatitis C virus (HCV) were negative. He was not immunized against the hepatitis B virus (HBV).

**Table 1:** Summary of laboratory findings ACPAs: antibodies to citrullinated protein antigens; ALP: alkaline phosphatase; ALT: alanine aminotransferase; ANAs: anti-nuclear antibodies; ANCA: anti-neutrophil cytoplasmic antibodies; anti-dsDNA: anti-double-stranded DNA; APLAs: antiphospholipid antibody; AST: aspartate aminotransferase; C3: complement component 3; C4: complement component 4; CK: creatine kinase; CRP: C-reactive protein; ENA: extractable nuclear antigens; ESR: erythrocyte sedimentation rate; GGT: gamma-glutamyl transferase; IgA: immunoglobulin A; IgG: immunoglobulin G; IgM: immunoglobulin M; LDH: lactate dehydrogenase; RF: rheumatoid factor.

Parameter	Value	Reference value	Parameter	Value	Reference value
Hemoglobin	11.2 g/dL	13.0-18.0 g/dL	Albumin	42.4 g/L	38.0-51.0 g/L
Leucocytes	10.31x10^9^/L	4.0-10.0x10^9^/L	ESR	68 mm/1^st^ hour	0-15 mm/1^st^ hour
Neutrophils	73.2%	53.8-69.8%	LDH	118 U/L	135-225 U/L
Lymphocytes	15.5%	22.6-36.6%	CK	84 U/L	10-172 U/L
Eosinophils	3.9%	0.6-4.6%	Myoglobin	75 ng/mL	<146.9 ng/mL
Platelets	371x10^9^/L	150-450x10^9^/L	Microalbuminuria (24-hour urine collection)	144.4 mg/24h	<30.0 mg/24h
CRP	44.6 mg/L	<3.0 mg/L	Total proteins (24-hour urine collection)	0.91 g/24h	<0.10 g/24h
Creatinine	2.24 mg/dL	0.67-1.17 mg/dL	ANAs	1/100 (homogeneous pattern)	<1/100
Urea	60 mg/dL	10-50 mg/dL	Anti-dsDNA antibodies	<10.0 UI/mL	<100 UI/mL
Sodium	137 mEq/L	135-147 mEq/L	ANCA panel	<2 U/mL	<20 U/mL
Potassium	4.0 mEq/L	3.5-5.1 mEq/L	ENA antibodies panel	Negative	
Chloride	103 mEq/L	101-109 mEq/L	C3	173 mg/dL	83.0-177.00 mg/L
Ionized calcium	1.34 mmol/L	1.22-1.36 mmol/L	C4	37 mg/dL	12-36 mg/dL
ALT	14 U/L	10-37 U/L	Rheumatoid factor	<11.3 UI/mL	<30.0 UI/mL
AST	13 U/L	10-37 U/L	ACPAs	0.9 U/mL	<7.0 U/mL
GGT	35 U/L	10-49 U/L	APLAs	<3 GPL/MPL	<20.0 GPL/MPL
ALP	109 U/L	30-120 U/L	IgG	1650 mg/dL	650-1500 mg/dL
Total bilirubin	0.43 mg/dL	<1.20 mg/dL	IgA	416 mg/dL	78-312 mg/dL
Direct bilirubin	0.16 mg/dL	<0.40 mg/dL	IgM	110 mg/dL	55-300 mg/dL

Renal ultrasound showed preserved kidney dimensions, without asymmetries and without hydronephrosis. Chest X-ray was unremarkable, without hilar lymphadenopathy or pulmonary cavities.

The patient was admitted to the Internal Medicine Department. To clarify this AKI, further investigation was performed (see Table 1 for details). He had a mild elevation of the erythrocyte sedimentation rate and analysis of 24-hour urine collection showed microalbuminuria (144.4 mg/24 h). To exclude an autoimmune process as a contributor to the AKI given the history of anemia and UC, we requested an immunological study that revealed positive anti-nuclear antibodies with a titer of 1/100 in a homogeneous pattern, while anti-double-stranded DNA antibodies were negative and there was no evidence of complement consumption. The remaining immunological study was negative. Immunoglobulin (Ig) quantification showed a mild elevation of both serum IgG (with IgG subclasses within normal range) and IgA. Seric and urinary immunoglobulin free light chains ratio were within normal range and serum protein electrophoresis analysis did not suggest monoclonal gammopathy. Blood and urine cultures did not grow any microbiological agent. Serological tests for cytomegalovirus, parvovirus B19, herpes simples 1&2, varicella-zoster virus, measles, rubella, *Toxoplasma gondii*, and *Treponema pallidum* particle agglutination assay (TPPA) were negative.

It was hypothesized that this patient’s AKI was due to an extraintestinal manifestation of UC or resulted from an adverse effect of mesalazine therapy. To clarify this, a colonoscopy was requested, which showed colonic mucosa with loss of normal vascular pattern, erythema, friability, and erosions (Mayo subscore 2). A renal biopsy was performed and revealed an interstitial lymphoplasmacytic infiltrate with multifocal tubulitis and scattered eosinophils compatible with acute interstitial nephritis (AIN); an immunofluorescence study verified the absence of deposits.

It was decided to discontinue treatment with mesalazine, and the patient was started on corticosteroid therapy with prednisolone at 1 mg/kg/day with improvement in kidney function to baseline levels in subsequent evaluations.

The patient was discharged with the indication to maintain corticosteroid therapy at a dose of 1 mg/kg/day for two weeks followed by a taper of 10 mg per week over eight weeks. During weaning off corticosteroid therapy, the patient was reassessed at the nephrology clinic and displayed sustained improvements in renal function, with return to pre-admission serum creatinine levels.

**Figure 1:**
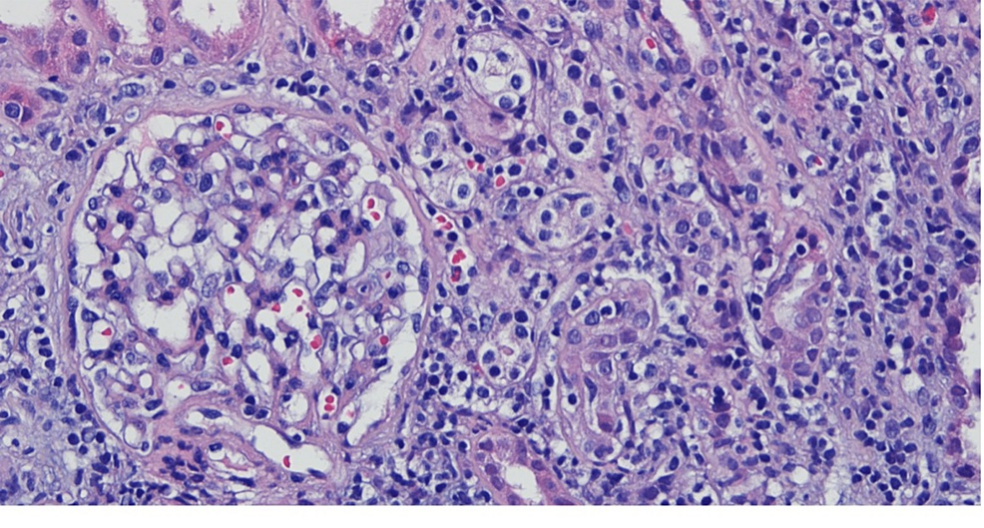
Renal biopsy On renal biopsy, it is possible to observe tubulitis and lymphoplasmacytic infiltrate with eosinophils in the renal interstitium.

Taking into account our patient’s history, these lesions were most likely due to mesalazine (Figure 1).

## Discussion

AIN is an important cause of AKI, causing nearly 30% of unexplained cases of renal impairment in adults. Its prevalence is rising due to increasing polypharmacy in older adults. Etiologies for AIN include drugs (70-75%), systemic diseases such as sarcoidosis, Sjogren syndrome and systemic lupus erythematous (10-20%), infections (4-10%), and tubulointerstitial nephritis with uveitis (less than 5%). Many cases of AIN result from exposure to antibiotics (mainly beta-lactam antibiotics), but virtually any drug can cause AIN [1,2]. Drug-induced AIN (DI-AIN) occurs in an idiosyncratic and non-dose-dependent manner and re-occurs on re-exposure. There is no typical range of time of onset for DI-AIN [1,2]. When reviewing the literature, mesalazine was the most likely aminosalicylate associated with AIN [2-4].

We present this case to highlight how challenging it was to establish if the AIN was secondary to a drug reaction or an extraintestinal manifestation of inflammatory bowel disease (IBD). Between 4 and 23% of patients with IBD present with renal manifestations such as calcium oxalate nephrolithiasis, immune complex glomerulonephritis, AIN, and renal amyloidosis. However, AIN is rarely associated with UC [2,5].

## Conclusions

Patients may present with nonspecific signs of AKI like nausea, vomiting, and malaise, while many patients are asymptomatic. The classical presentation with rash, fever, and peripheral eosinophilia occurs in only 10% of patients and a definitive diagnosis of AIN requires a kidney biopsy.

Mesalazine should be withdrawn when there is an AKI in a patient with IBD. Besides discontinuation of the offending agent, corticosteroids are the best available treatment for AIN, especially when administered early after the diagnosis.

In summary, it is important that all patients being treated with mesalazine have an evaluation of kidney function prior to the initiation of the drug and periodically while on therapy (every 3-6 months for the first year and then annually), to assess for declines in kidney function that might be attributable to ongoing therapy.
